# Institutional Questions

**Published:** 1899-09-16

**Authors:** 


### INSTITUTIONAL QUESTIONS.

**Ward Tables and Lockers.**

"A Correspondent" writes: Kindly tell me what material you consider best for the tops of ward lockers, tables, &c.? I believe that glass is now much used instead of marble.

Yes; glass is for many reasons to be preferred to marble. It is cheaper, and, what is even more important, it is easier to clean and is not spoilt by acids or other chemicals as is marble. Nothing makes a more satisfactory top to lockers and tables than a sheet of plain glass, and, so far as appearances go, the effect is excellent.

**Hospital Walls.**

A "Correspondent" asks: Do you consider "cavity walls" necessary or desirable for hospital wards?

Solid walls are best for ward construction, unless there is some special reason, such as extreme porousness of the material to be used for buildings, or a very exposed site, to render cavity walls desirable. In the latter case care should be taken that the inner wall be at least nine inches thick, so as to secure the inter-space against any connection with the interior of the building. Otherwise the cavity may become a harbour for dirt, and so a source of danger rather than a protection.

**A Nurses' Association.**

"A Nurse" writes: In forming an association of trained nurses is registration necessary? What are its advantages, and where should one apply for definite information as to the steps to be taken?

Your question is somewhat vague, and gives no indication of the sort of "association" which it is proposed to form. We may point out, however, that if it is a private venture of your own there would seem to be no special object in incurring the expense of incorporation, which would amount to from £50 to £100. If a public association of nurses is contemplated, worked by a committee, then the need for incorporation would not arise until it is established and its plan of work definitely settled by experience. When that time arrives the services of a solicitor would be required to prepare the necessary papers for incorporating the association under the Board of Trade should such a step be decided upon.

**Patients' Clothes.**

"A Matron" asks: May I trouble you for advice on the following matter? Is it not an objectionable practice to keep patients' clothes in boxes or baskets under the beds in a ward? I am trying to obtain cupboards outside the wards to use for this purpose, but I find I am looked upon as "faddy" for desiring what is considered to be an "innovation."

Certainly you are perfectly right in wishing to banish clothes receptacles from under the beds in your wards. It is a most undesirable plan, and in all up-to-date institutions now quite given up in favour of special cupboards, which should be outside the ward, and even there should be specially well ventilated. They should be divided into compartments, numbered to correspond with the beds. Modern asepticism is gradually driving every unnecessary article beyond the ward door, and even an elementary knowledge of the first principles of hygiene will show reasons why such a plan as the one you very properly condemn should be abandoned.

**Slab or Table for Mortuary.**

"A Chairman of House Committee" writes: Kindly reply to the following query in your "Answers" column:—What is the best form of slab or table to place in a small cottage hospital mortuary, bearing in mind the possibility of post-mortem examinations being sometimes necessary? I believe such a slab should be grooved to carry off fluid matter. The floor will be concrete.

In the case referred to there are certain advantages in a table the top of which is constructed of marble, the principal one being that it can be somewhat "dished" so that any fluid will run to a central channel. At the Middlesex Hospital the mortuary is fitted with glass slabs, supported on white enamelled iron frames or stands. Similar slabs are there used in the post-mortem room, and one or more of these should suit all your needs. If the slab be dished there would be no need to have more than one deep groove down the middle of the slab, but for post-mortem purposes there should be a hole, beneath which a bucket could be placed to receive fluid if required. Porcelain is no doubt a better material than glass, but more expensive, and hardly necessary in the case of a small hospital, where post-mortem examinations would hardly take place more than two or three times a year. Plate glass is not necessary; rolled glass is the cheapest and most cleanly material possible, and far better than slate. The iron frames should be strongly made, heavy enough not to be easily moved, but not fixed to the floor, as in the event of a post-mortem it is desirable to have free space on all sides, and a change of position might be convenient. You will, of course, have water laid on, and a hose provided for the most efficient cleansing. The cost of such a table as that described is very small. For more elaborate fitments we can only refer you to the trade catalogues, but for your purpose these would scarcely be required.

